# Cheating on examinations and its predictors among undergraduate students at Hawassa University College of Medicine and Health Science, Hawassa, Ethiopia

**DOI:** 10.1186/1472-6920-14-89

**Published:** 2014-04-30

**Authors:** Anteneh Assefa Desalegn, Asres Berhan

**Affiliations:** 1 Pharmacology Unit, School of Medicine, Hawassa University, Hawassa, P.O Box-1560, Ethiopia

**Keywords:** Cheating, Lecture, Academic dishonesty, Higher institutions

## Abstract

**Background:**

Cheating on examinations in academic institutions is a worldwide issue. When cheating occurs in medical schools, it has serious consequences for human life, social values, and the economy. This study was conducted to determine the prevalence of cheating and identify factors that influence cheating among students of Hawassa University College of medicine and health science.

**Methods:**

A cross sectional study was conducted from May through June 2013. A pre-tested self-administered, structured questionnaire was used to collect self-reported data regarding cheating. Data were entered and analyzed using SPSS version 20. Descriptive statistics were used for data summarization and presentation. Degree of association was measured by Chi Square test, with significance level set at p = 0.05. Bivariate and multivariate logistic regression analyses were used to assess associations.

**Results:**

The prevalence of self-reported cheating was found to be 19.8% (95% CI = 17.4-21.9). About 12.1% (95% CI = 10.2-13.9) of students disclosed cheating on the entrance examination. The majority of students (80.1% (95% CI = 77.9-82.3) disclosed that they would not report cheating to invigilators even if they had witnessed cheating. Analysis by multiple regression models showed that students who cheated in high school were more likely to cheat (adjusted OR = 1. 80, 95% CI = 1. 01–3.19) and that cheating was less likely among students who didn’t cheat on entrance examinations (adjusted OR = 0. 25, 95% CI = 0. 14–0.45). Dining outside the university cafeteria and receiving pocket money of Birr 300 or more were strongly associated with cheating (adjusted OR = 3.08, 95% CI = 1.54-6.16 and adjusted OR = 1.69 (95% CI = 1.05-2.72), respectively. The odds of cheating among students were significantly higher for those who went to private high school, were substance users, and didn’t attend lectures than for those who attended government schools, were not substance abusers, and attended lectures.

**Conclusion:**

Our findings have important implications for development of an institution’s policies on academic integrity. By extension, they affect the policies of high schools. Increased levels of supervision during entrance examination, mandated attendance at lectures, and reduction of substance use are likely to reduce cheating. No significant association was found with background, level of parental education, grade point average, and interest in field of study.

## Background

The predictors of cheating vary in different societies and cultural backgrounds. Regardless, cheating in medical and health science institutions should not be tolerated because it has serious consequences on human lives, social values, and economy. Academic cheating is defined as being found in possession or copying from materials brought in to an exam that are not specifically permitted or allowing a student to copy from one’s exam paper through oral, symbolic, written and electronic or any other means. Academic cheating has become an area of concern in university education worldwide. Hawassa University College of Medicine and Health Science is not an exception. The goals of learning put forth by Ministry of education in Ethiopia or elsewhere are in jeopardy because what students learn is finally measured by different assessment techniques. Cheating is one of the main factors which leads to faulty assessment and hence renders a false message on evaluation of students [1-3].

Different research on cheating confirms widespread and prevalent academic dishonesty in academic institutions in the world and shows that some types of cheating have increased considerably in the past decades [4-7]. In fact, cheating has become an almost common phenomenon and with particularly technology becoming more sophisticated every day, academic dishonesty in universities is increasing and has become a worldwide issue [7].

According to a multicampus investigation of the relationship between academic dishonesty and workplace dishonesty, “There is general agreement that there should be zero tolerance of cheating in a profession based on trust and one on which human lives depend. It is reasonable to assume that cheaters in medical school will be more likely than others to continue to act dishonestly with patients, colleagues, insurers, and government” [8]. Cheating is a very serious problem not only because it affects the quality of the education system, but it is also unfair to those who don’t cheat. In addition, it gives a false message on evaluation of students’ knowledge and skill. It can cause damage to the society as it results in professionals with poor quality. And when this occurs in medical and health science professionals, it costs human life and has an economic impact. In addition, studies have shown that academic dishonesty at college can also be a predictor of workplace dishonesty [9].

The available literature shows that predictors of cheating are multi-factorial. First of all, values acquired long before joining the university such as cultural, religious, moral, social and familial could determine current cheating behavior [8,10]. Additional factors that could influence decision after students join a university include honor codes of institutions, academic overload, peer norm, classroom environment, cumulative grade point average and anticipated reward [4,11-13] Other factors include a positive attitude toward cheating, prior cheating behavior, year of study, gender and technology [7,10,13-18].

The objective of this study was to determine the prevalence of cheating and identify predictors of cheating among students of Hawassa University College of Medicine and Health Science. Based on our finding, we intend to provide baseline evidence for policy makers of this institution that will allow them develop academic environment that promotes integrity and consequently restores and preserves trust in the health professionals of our institution and our entire country.

## Methods

### Study Design and Setting

From May 2013 to June 2013, we conducted a cross-sectional study using a pre-tested self administered structured questionnaire on self reported accounts of cheating to fulltime undergraduate students of Hawassa University College of Medicine and Health Science, Ethiopia. The College of medicine and health sciences was established in 2003 and consists of three schools (Medicine; Public health and Environmental sciences; Nursing and Midwifery) and the department of Medical Laboratory Sciences with 7 undergraduate programs.

### Eligibility Criteria and Data Collection

This study included all regular undergraduate students who stayed in the college for at least one semester and consented to participate in the study. There were 1,366 eligible fulltime undergraduate students from a total of 1,732 undergraduate and postgraduate students. A self administered pre-tested structured English questionnaire was used for data collection. The questionnaire was pre-tested on part time undergraduate health science students before the data collection. In parts of the questionnaire that were found to be misleading or confusing, a slight modification was made on the wording before data collection. Almost all questions were close-ended with pre-coded response. The questionnaire had two categories: 1) demographic and socio-cultural characteristics and 2) reasons for cheating and not cheating during examination. Trained data collection facilitators distributed the questionnaires to study participants in lecture halls, classrooms, and dormitories. To assure anonymity, the study participants were told not to put identifying information on the completed questionnaires, to seal them, and to place them in data collection box.

### Measurement of Cheating

The dependent variable for this study was the act of cheating as self reported. The independent variables included sex, age, cultural background, parents’ education level, religious belief, cumulative GPA, workload of students, the experience of cheating at high school, type of high school attended, perception of peers to cheating, competition with peers, social drug use, class size and year of study. The socio-cultural variables are important because some students in our university live and dine outside the campus where they lead a different life style from the majority. These changes have been introduced recently in our University. We believed it might have either a positive or a negative effect on their behavior. Thus, we wanted to see how independent variables such as “Students’ residence” and “location of eating meal” affected cheating.

### Statistical Analysis

All the gathered data from the students was entered, cleaned, coded, and analyzed using SPSS version 20 software (SPSS Inc, Chicago, IL, USA). Descriptive statistics were used for data summarization and presentation. Degree of bivariate association was measured by using the Pearson Chi square test to assess the significance between different variables, with a significance level set at p = 0.05. Finally, a multivariate analysis was done by fitting the logistic regression model to identify risk factors associated with committing an act of cheating during exams and reporting observed act of cheating by controlling for the effect of potential confounding variables such as age, sex, religion, rural–urban background, parental education, student’s monthly income from family, type of high school attended, cumulative GPA, workload of students, experience of cheating at high school, perception of peers to cheating, competition with peers, social drug use, class size, field of study and year of study.

### Ethical Clearance

The Institutional review board of Hawassa University College of Medicine and Health Science approved this study. Participation in the study was voluntary and the purpose of the study was explained to students prior to distribution of the questionnaires. Written consent was obtained from each study participant by attaching a statement of consent to each questionnaire. However, identification of the students was not recorded anywhere on the questionnaire and confidentiality was assured by analyzing the data in aggregate.

## Results

Of 1,366 eligible regular undergraduate medical and health science students, 1,119 (81.9%) provided consent and completed the questionnaire. The main reason for non-participation was unavailability of students at the time of the survey at lecture halls, classroom or dormitory and clinical or community based attachment of students outside Hawassa University college of Medicine and health sciences. In addition, only students who stayed more than a semester were allowed to participate.

The demographic and socio-cultural characteristics of the study participants are presented in table 1. More men (78.8%) than women answered the survey. The overall age of students ranged from 18 to 35 years, with the majority (74.2%) being between 20 to 24 years. The mean age of the students was 21.5 years (SD =1. 902 years), and currently the average number of students per class is 83.4 (SD = 46.8) with a minimum of 14 students per class and a maximum of 255 students per class. Of the total participants, 682 (57.7%) had grown-up in urban areas. The majority (58.4%) of students were orthodox Christians, followed by Protestant Christian (26.4%) and Muslim (11.2%). The majority (79.1%) of students attended government or private high school before they joined the university. A total of 424 (35.1%) students studied medicine. Other programs of study were public health (23.2%), nursing (15.7%), midwifery (10%), medical laboratory (8%) and optometry (5.1%).

**Table 1:** Socio-demographic characteristics of the students by sex, June 2013

**Characteristics**	**Sex**		**Total, n (%)**
	**Male, n (%)**	**Female, n (%)**	
**Age in years**			
15-19	91(7.5%)	45(11.2%)	136(11.2%)
20-24	735(60.3%)	170(13.9%)	905(74.2%)
25 or more	134(11%)	44(3.6%)	178(14.6%)
**Original background**			
Urban	491(41.5%)	191(16.1%)	682(57.7%)
Rural	447(37.8%)	54(4.6%)	501(42.3%)
**Religion**			
Christian (protestant)	256(21.1%)	64(5.3%)	320(26.4%)
Christian (Orthodox)	557(46%)	151(12.5%)	708(58.4%)
Muslim	103(8.5%)	33(2.7%)	136(11.2%)
Other	38(3.1%)	10(0.8%)	48(4%)
**Type of high school attended**			
Public school	814(67.6%)	139(11.5%)	953(79.1%)
Private school	119(9.9%)	107(8.9%)	226(18.8%)
Missionary school	15(1.2%)	11(0.9%)	26(2.2%)
**Mother’s education level**			
Illiterate	374(31.3%)	37(3.1%)	411(34.4%)
Primary education	280(23.4%)	51(4.3%)	331(27.7%)
Secondary education	147(12.3%)	53(4.4%)	200(16.7%)
College diploma	82(6.9%)	69(5.8%)	151(12.6%)
First degree or above	60(5%)	42(3.5%)	102(8.5%)
**Father’s education level**			
Illiterate	274(22.9%)	27(2.3%)	301(25.2%)
Primary education	256(21.4%)	37(3.1%)	293(24.5%)
Secondary education	151(12.6%)	40(3.3%)	191(16%)
College diploma	132(11%)	39(3.3%)	171(14.3%)
First degree or above	129(10.8%)	110(9.2%)	239(20%)
**Location of student’s residence**			
In the university dormitory	900(74.4%)	220(18.2%)	1120(92.6%)
Outside the university campus	42(3.5%)	14(1.2%)	56(4.6%)
With family	13(1.1%)	20(1.7%)	33(2.7%)
**Location of eating meal**			
At the university’s café	876(72.7%)	93(7.7%)	969(80.4%)
Outside the university café	76(6.3%)	160(13.3%)	236(19.6%)
**Monthly Pocket money**			
Less than 100 birr	105(8.8%)	9(0.8%)	114(9.6%)
100-299 birr	434(36.4%)	52(4.4%)	486(40.8%)
300-500 birr	291(24.4%)	121(10.2%)	412(34.6%)
More than 500 birr	112(9.4%)	68(5.7%)	180(15.1%)
**Field of study**			
Nursing	133(11%)	56(4.6%)	189(15.7%)
Midwifery	94(7.8%)	27(2.2%)	121(10%)
Medical laboratory technology	77(6.4%)	19(1.6%)	96(8%)
Public health officer	234(19.4%)	46(3.8%)	280(23.2%)
Medicine	340(28.2%)	84(7%)	424(35.1%)
Optometry	43(3.6%)	191.6%)	62(5.1%)
Environmental health officers	33(2.7%)	2(0.2%)	35(2.9%)
**Year of study**			
1st year	100(8.2%)	37(3%)	137(11.3%)
2nd year	293(24.1%)	64(5.3%)	357(29.4%)
3rd year	216(17.8%)	68(5.6%)	284(23.4%)
4th year	276(22.7%)	75(6.2%)	351(28.9%)
5th year	74(6.1%)	13(1.1%)	87(7.2%)
**Total, N (%)**	960(78.8%)	259(21.2%)	1219(100%)

Table 2 summarizes the perception and self reported prevalence of cheating by students at high school, during the university entrance examination and while currently enrolled at Hawassa University. The current prevalence of cheating in the university among students was reported to be 19.8% (95% CI, 17.4, and 21.9), slightly lower than high school 21.2% (95% CI = 17.4-21.9). A greater number of females than males reported cheating in high school and university, showing a statistically significant difference (p = 0.001 and p = 0.005, respectively). About 12.1% (95% CI = 10.2-13.9) of the students reported cheating on the university entrance examination and again, more females (16.2%) than males (11%) cheated on entrance examination (p = 0.027). In general, female students were more likely to cheat in high school, on the entrance examination and while attending the university.

**Table 2:** Perception and reported cheating account by students at high school, on entrance examination and in the university by sex, June 2013

**Act of cheating and perception towards cheating**	**Sex**		**Total, n (%)**	**P-value**
	**Male, n (%)**	**Female, n (%)**		
During high school				**0.001**
Yes	177(19.2%)	68(28.7%)	245(21.2%)	
No	743(80.8%)	169(71.3%)	912(78.8%)	
At entrance examination				
Yes	102(11%)	39(16.2%)	141(12.1%)	**0.027**
No	827(89%)	202(83.8%)	1029(87.9%)	
Currently at the university				**0.005**
Yes	166(18.1%)	63(26.2%)	229(19.8%)	
No	751(81.9%)	177(73.8%)	928(80.2%)	
Allowed others to cheat				0.627
Yes	285(30.2%)	80(31.7%)	365(30.5%)	
NO	660(69.8%)	172(68.3%)	832(69.5%)	
Considered as normal among classmates				**0.02**
Yes	193(20.5%)	68(27.4%)	261(22%)	
No	747(79.5%)	180(72.6%)	927(78%)	
Report to invigilators if witnessed				0.277
Yes	191(20.5%)	43(17.4%)	234(19.9%)	
NO	740(79.5%)	204(8%)	944(80.1%)	
Is cheating a common practice				**0.014**
Yes	199(22.9%)	64(31.1%)	263(24.4%)	
NO	671(77.1%)	142(68.9%)	813(75.6%)	

Note: P-values in bold are statistically significant (p < 0.05).

Regarding perception of students toward cheating, of the study participants, 22% (95% CI = 19.5-24.4) perceived that their peers thought cheating to be an acceptable behavior or normal phenomenon (27.4% females vs. 20.5% males, p = 0.02). About one in four (24.4% (95% CI = 21.9-27)) perceived cheating to be common or very common practice among classmates (31.1% females vs. 22.9% males, p = 0.014). About 14.8% (95% CI = 12.8-16.9) perceived cheating to occur only sometimes among class mates whereas 53.4% believed it occurred very rarely or rarely or never. 30.5% (95% CI = 27.7-31.1) of study participants admitted to passive cheating, that is allowing others to copy or cheat from them. The majority (78% (95% CI = 75. 6–80.5)) of the students of Hawassa University College of Medicine and health science regard cheating in exam as unacceptable behavior, however 80.1% (95% CI = 77. 9–82.3) disclosed that they would not report cheating to invigilators if they witnessed an act of cheating (Table 2).

Table 3 summarizes data regarding reported cheating by different characteristics. About one in five students at the university cheated. A significant number of females reported cheating as compared to males (26.2% females vs. 18.1% males, p = 0.005). Students with an urban background cheated more than those from a rural background (23.8% urban vs. 14.3% rural, p < 0.001). Of the study participants, 28.7% did not study in the field of their choice at the university, and students who did not study in their chosen field of study cheated more (26.5%) than those who did (17.1%) (p < 0.001). On the other hand, 289 (25%) of the students reported that they were not interested in their field of study. The students who did not like their field of study also cheated more than those who were interested in their field of study (26% vs. 17.7%, p = 0.002). The prevalence of substance use among students was 13.5%, a significant number of students who engaged in substance abuse reported cheating (41.2%) as compared with those who didn’t cheat (16.3%) (p < 0.001). Students who were absent from one or more lectures cheated more (21.2%) than those who never missed lecture classes (16.1%).

**Table 3:** Reported ever cheating by students currently at the university by different characteristics, June 2013

**Characteristics**	**Ever cheating**		**Total**	**P-Value**
	**No**	**Yes**		
**Gender**				
Male	751(81.9%)	166(18.1%)	917(79.3%)	**0.005**
Female	177(73.8%)	63(26.2%)	240(20.7%)	
**Students' background**				
Urban	491(76.2%)	153(23.8%)	644(57.2%)	**<0.001**
Rural	413(85.7%)	69(14.3%)	482(42.8%)	
**Joined field of study by choice**				
Yes	685(82.9%)	141(17.1%)	826(71.3%)	**<0.001**
No	244(73.5%)	88(26.5%)	332(28.7%)	
**Interest on/Like field of study**				
Yes	712(82.3%)	153(17.7%)	865(75%)	**0.002**
No	214(74%)	75(26%)	289(25%)	
**Substance use**				
No	820(83.7%)	160(16.3%)	980(86.5%)	**<0.001**
Yes	90(58.8%)	63(41.2%)	153(13.5%)	
**Attended all lecture classes**				
Yes	240(83.9%)	46(16.1%)	286(25.1%)	0.06
No	673(78.8%)	181(21.2%)	854(74.9%)	

Note: P-values in bold are statistically significant (p < 0.05).

**Figure 1:**
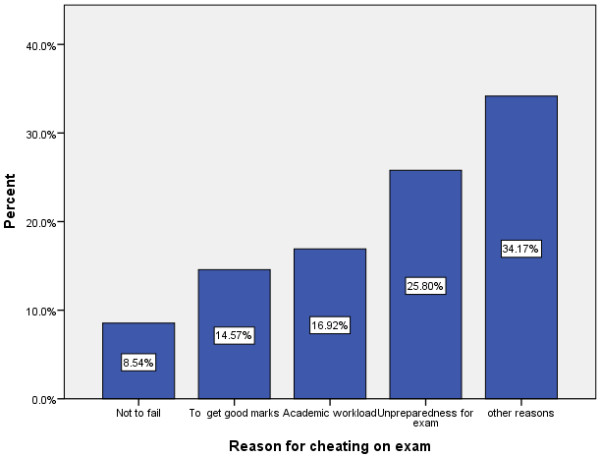
Reasons for cheating report by students.

**Figure 2:**
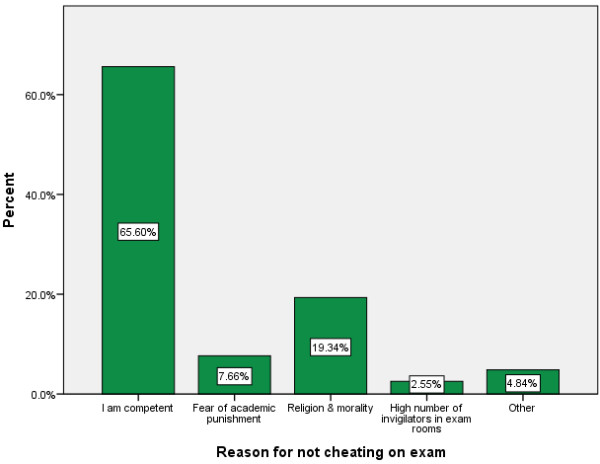
Reasons for not cheating on exams as reported by students.

Figure 1 depicts the reasons for cheating while Figure 2 depicts reasons for not cheating as reported by students. The major reasons reported by students for cheating on exams were lack of preparation for exams (25.7%), academic workload or other assignments (16.9%), need to obtain good marks (14.6%), desire not to fail exams (8.6%) and about 34% mentioned other reasons. On the other hand, the main reasons given by students for not cheating on exams are feeling competent (65.6%), being true to religious and moral standards (19.3%), fear of academic punishment (7.7%), presence of strict invigilators (2.6%) and other reasons (4.8%). The major means used for cheating as reported by students are symbolic (28.74%), oral (26.82%), written (23.37%) and electronic methods (9.96%).

Table 4 summarizes socio-demographic and behavioral variables of study participants who acknowledged that they cheat. Variables were assessed using logistic regression. The odds of cheating among students who went to private high school were significantly higher than for students who went to public or governmental schools (adjusted OR = 1.80, 95% CI = 1.01-3.19). Cheating was strongly associated with students who dine outside the university cafeteria as compared with those who dined at the university cafeteria (adjusted OR = 3.08, 95% CI = 1.54-6.16). Students who received a monthly income or pocket money of Birr 300 or more were more likely to cheat as compared to those students who received fewer than Birr 300 (adjusted OR = 1. 69 (95% CI = 1. 05–2.72). Cheating was associated with field of study. Compared to nursing students, the odds of cheating for medical students was 84% lower (adjusted OR = 0. 16, 95% CI, 0.07-0.34), 72% (adjusted OR = 0. 28, 95% CI = 0. 09–0.83) lower for optometry students, and 71% (adjusted OR = 0. 21, 95% CI = 0. 13–0.67) lower for midwifery students.

**Table 4:** Socio-demographic and behavioral correlates of current cheating among Hawassa University College of medicine and health science students, June 2013

**Factors**	**Ever cheating**		**Adjusted OR (95% CI)**	**p-value**
	**No**	**Yes**		
**Gender (N = 1157)**				
Female	177(73.8%)	63(26.2%)	0.73(0.38,1.39)	0.344
Male	751(81.9%)	166(18.1%)	1.00(Reference)	
**Original background (N = 1126)**				
Urban	491(76.2%)	153(23.8%)	1.00(Reference)	0.858
Rural	413(85.7%)	69(14.3%)	0.95(0.58,1.55)	
**Entrance exam score range (N = 1070)**				
Excellent	199(84.3%)	37(15.7%)	1.00(Reference)	
Very good	398(79.8%)	101(20.2%)	0.91(0.51,1.63)	0.768
Good	233(76.9%)	70(23.1%)	0.85(0.44,1.61)	0.625
Fair	17(81%)	4(19%)	0.33(0.50,2.32)	0.270
Poor	7(63.9%)	4(36%)	1.05(0.19,5.83)	0.950
**Type of high school attended (N = 1146)**				
Public school	764(84.1%)	144(15.9%)	1.00(Reference)	
Private school	139(65.3%)	74(34.7%)	1.80(1.01, 3.19)	**0.045**
Missionary school	18(72%)	7(28%)	2.4(0.61,9.94)	0.204
**Mother’s education level (N = 1143)**				
Illiterate	339(87.1%)	50(12.9%)	1.00(Reference)	
Literate	578(76.7%)	176(23.3%)	0.85(0.46,1.58)	0.622
**Father’s education level (N = 1143)**				
Illiterate	246(86.3%)	39(13.7%)	1.00(Reference)	
Literate	670(78.1%)	188(21 > 9%)	0.92(0.48,1.76)	0.823
**Residence (N = 1156)**				
In the university	874(81.7%)	196(18.3%)	1.00(Reference)	
Rental house	39(72.2%)	15(27.8%)	1.05(0.406,2.72)	0.916
With family	15(46.9%)	17(53.1%)	1.19(0.40,3.48)	0.748
**Dining place of students (N = 1152)**				
At the university’s café	775(83.6%)	152(16.4%)	1.00(Reference)	
Outside the university café	149(66.2%)	76(33.8%)	3.08(1.54,6.16)	**0.001**
**Monthly Pocket money (N = 1141)**				
Less than birr 300	489(85.3%)	84(14.7%)	1.00(Reference)	
More than birr 300	426(75%)	142(25%)	1.69(1.05,2.72)	**0.028**
**Field of study(N = 1155)**				
Nursing	122(66.3%)	62(33.7%)	1.00(Reference)	
Midwifery	99(86.8%)	15(13.2%)	0.29(0.13,0.67)	**0.004**
Medical laboratory technology	74(80.4%)	18(19.6%)	0.51(0.22,1.19)	0.122
Public health officer	192(72.2%)	74(27.8%)	0.86(0.47,1.55)	0.620
Medicine	368(91.3%)	35(8.7%)	0.16(0.07,0.34)	**<0.001**
Optometry	48(78.7%)	13(21.3%)	0.28(0.09,0.83)	**0.022**
Environmental health officers	23(65.7%)	12(34.3%)	1.24(0.46,3.37)	0.664
**Year of study(N = 1158)**				
1st year	82(62.1%)	50(37.9%)	1.00(Reference)	
2nd year	283(81.3%)	65(18.7%)	0.51(0.26,1.01)	0.056
3rd year	218(80.1%)	54(19.9%)	0.52(0.26,1.02)	0.058
4th year	270(83.1%)	55(16.9%)	0.25(0.12,0.53)	**<0.001**
5th year	76(93.8%)	5(6.2%)	0.45(0.11,1.78)	0.256
**Choose their field of study (N = 1158)**				
Yes	685(82.9%)	141(17.1%)	1.00(Reference)	
No	244(73.5%)	88(26.5%)	1.04(0.62,1.74)	0.879
**Like their field of study (N = 1154)**				
Yes	712(82.3%)	153(17.7%)	1.00(Reference)	
No	214(74%)	75(26%)	1.31(0.78,2.20)	0.295
**Consider cheating normal (N = 1142)**				
Yes	179(70.8%)	74(29.2%)	1.00(Reference)	
No	736(82.8%)	153(17.2%)	1.01(0.61,1.64)	0.969
**Allowed others to cheat or copy (N = 1150)**				
Yes	225(64.5%)	124(35.5%)	1.00(Reference)	
No	697(87%)	104(13%)	0.45(0.29,0.70)	**<0.001**
**Cheated on entrance examination (N = 1146)**				
Yes	58(42.6%)	78(57.4%)	1.00(Reference)	
No	867(85.8%)	144(14.2%)	0.25(0.14,0.45)	**<0.001**
**Attending all lecture class (N = 1140)**				
Yes	240(83.9%)	46(16.1%)	1.00(Reference)	
No	673(78.8%)	181(21.2%)	1.74(1.04,2.91)	**0.033**
**Use of substance (N = 1133)**				
NO	820(83.7%)	160(16.3%)	1.00(Reference)	
Yes	90(58.8%)	63(41.2%)	2.16(1.23,3.81)	**0.007**
**Cheated at high school (N = 1140)**				
No	786(87.1%)	116(12.9%)	1.00(Reference)	
Yes	133(55.9%)	105(44.1%)	2.96(1.83,4.78)	**<0.001**

Note: P-values in bold are statistically significant (p < 0.05).

Cheating was also significantly associated with years of study, and the odds of cheating decreasing with increasing years of study. The odds of cheating among fourth year students was 75% lower than first year students (adjusted OR = 0.25, 95% CI = 0.12-0.53). Not attending all or most lecture classes was strongly and positively associated with cheating (adjusted OR = 1.74, 95% CI = 1.04-2.91). Students who didn’t allow others to copy or cheat exams from them were less likely to cheat themselves (adjusted OR = 0.25, 95% CI = 0.14-0.45). The odds of cheating among students who didn’t cheat on the entrance examination to university was significantly lower than those who cheated (adjusted OR = 0. 25, 95% CI = 0. 14–0.45). The odds of cheating among students who cheated in high school was 2.9 fold higher than those who didn’t (adjusted OR = 2.96, 95% CI = 1.83-4.78). Substance use was also strongly associated with cheating among the study participants (adjusted OR = 2.16, 95% CI = 1.23-3.81).

## Discussion

This study showed that cheating is a common problem among undergraduate students of Hawassa University College of medicine and health science. The prevalence of self reported accounts of cheating by students was 19.8% (95% CI = 17.4-21.9). Such prevalence of cheating especially in college of medicine and health sciences should be unacceptable as it reflects the ethics of our future health care providers. This was evident in studies that have shown academic dishonesty is to be a good predictor of work place dishonesty [9]. Of the study participants, 21.2% (95% CI = 18.8-23.6) admitted cheating at high school. This is an equivalent percentage with the current cheaters in the university. This might show that student’s past experience of cheating at high school to be a good determinant of the practice of cheating latter in the university. In addition, 12.1% (95% CI = 10.2-13.9) of students admitted cheating in the entrance examination before they just joined the university (16.2% females, 11% males, p-value = 0. 027). And therefore, the same students might have continued to cheat in order to survive in the university. Similar studies in US have reported a prevalence of cheating among US medical students ranging from 0% to 58% [15].

The prevalence of reported cheating differed by discipline: Environmental health officers (34.3%), Nursing (33.7%), Public health officers (27.8%), Optometry (21.3%), medical laboratory technologists (27.8%), and medical (8.7%). Of the study participants, medical students seemed to be the most honest. In general, the prevalence shows that cheating has become an acceptable behaviour and prevalent among our study participants. The prevalence of cheating was higher than shown in a study done on second-year medical students in 31 schools in the US in 1991. The study showed the prevalence of cheating to be about 5% as compared with 8.7% reported for study participant who were medical students. The same study revealed that 40.5% of medical students cheated in high school, a higher proportion than the rate reported by than among currently enrolled medical students (19.3%) who participated in the study [6]. There is a big difference between cheating at high school and medical schools unlike our study where equivalent prevalence of cheating was observed both at high school and the university. This might be due to lack of strict invigilation practice during entrance examination to the university in our setup. A greater number of females than males reported cheating in high school and while currently at the university, this difference was statistically significant (p = 0.001 and p = 0.005, respectively). Contrary to our finding, many other studies [10,17] showed that self-reported cheating was more common in men than women.

30.5% (95% CI = 27.7-33.1) of study participants admitted to passive cheating (allowing their peers to copy exams or cheat from them). About 22% (95% CI = 19. 5–24.4) of study participants perceived cheating as an acceptable and normal behavior among their classmates (27.4% females vs. 20.5% males, P = 0.02). Other studies have also shown a positive correlation between cheating in high school and the belief position that cheating is morally acceptable [14]. A review of decades of research on cheating in academic institutions found that students' perception of peers’ behavior was the most powerful influence in the decision as whether or not to cheat [4]. A multidisciplinary exploration of college students’ perception of academic dishonesty also revealed that many students now see such behaviors as morally acceptable [19]. 24.4% (95% CI = 22. 1–26.8) viewed cheating as a common practice among their peers (31.3% females vs. 22.9% males, p = 0.014). And cheating amongst students is an almost common phenomenon with growing technology. Thus, academic dishonesty in universities is increasing and has become a worldwide issue [7].

Unpreparedness for exam, academic workload, getting good marks, and not to fail in exams were the major reasons reported by our study participants for cheating. Different studies have also reported similar reasons. A study at Texas dental hygiene schools identified academic overload as the primary explanation for cheating behaviour. Also the majority of respondent believed that it was necessary to cheat in order to get ahead and compete with their peers [20]. Other studies reported anticipation of reward for success, low grade point average, burnout and other reasons as justification for cheating [10,13,15]. Symbolic, oral, wriiten and electronic technologies were employed as a means of cheating among our study participants. Studies show that academic dishonesty to have a positive correlation with increased use of technology [7,18].

The majority (78%, 95% CI = 75. 6–80.5)) of our study participants regarded cheating in exam as unacceptable behavior. Similarly, in Israel, a study among medical students at Ben-Gurion University found that 73% of students to regarded cheating as unacceptable [14]. In contrast, a cross-sectional study at Croatian medical schools found that academic dishonesty was seen as an acceptable behaviour that 97% of the students admitted to form of cheating [21]. Students who believe that cheating was acceptable were more likely to cheat [9].

In our study, the vast majority of study participants (80.2%, 95% CI = 77.9-82.4) reported that they never cheated. Being competent, religious and moral beliefs, and fear of academic punishment were their major reasons not to engage in cheating. Studies showed that there was a distinct negative relation between students' estimated grade-point average and the tendency to engage in cheating behavior [13,22]. So the more competent a student is, there is no need to engage n cheating behavior. Other studies also shared some of the major reasons for not engaging in cheating practice. For example, a positive correlation was found between religious conviction and the belief that cheating is a morally negative practice [14]. Students with less firm religious or moral beliefs approved cheating more than did students with strong religious or moral convictions [23]. According to a survey on cheating among American and Japanese college students in 1994, the most effective deterrent to cheating was fear of punishment [11]. The level of supervision during exams and academic policies such as honor codes had also significant effects on cheating [4,23].

Of study participants, 80.1% (95% CI = 77.9-82.3) disclosed that they would not report cheating to invigilators even if they witnessed cheating. Other findings also showed that students refused to report acts of cheating because of interpersonal relationships [11,24].

The results from this study provide important insights into the magnitude of cheating and its associated predictors. The present study found that cheating in high school, cheating on the entrance examination for a University, passive cheating, field, and year of study were important factors very strongly associated with current cheating practice at the university.

We found that those study participants who reported cheating in high school and on entrance examinations were more likely to cheat while currently attendng the university. Previous studies also showed that cheating in high school was strongly associated with cheating at the university. A survey of second year students at 31 schools in the US found that the best predictor of whether someone would cheat in medical school was whether that person had cheated in high school [6]. Other surveys also indicated that students who cheated during high school years were more likely to cheat after entering college or university [10,14,15,25].

The results of our study showed that years of study were also negatively associated with cheating: Fourth-year students were less likely to cheat than first year students. Similarly, different studies confirmed that cheating was more common in younger students than mature ones [13,16,17]. In addition, in our study medical, midwifery and optometry students were less likely to cheat than nursing students. Also, study participants who allowed or helped others to copy (passive cheating) were more likely to be involved in active cheating. Findings from other studies showed that values such as friendship and interpersonal relationships could affect attitudes toward cheating [26] and that those individuals with a positive attitude toward cheating were also likely to engage in both passive and active cheating. In turn, those with positive attitudes toward cheating are more likely to engage in active cheating [4,9,10,12].

Our study found that substance abuse and eating meals outside the university cafeteria were strongly associated with cheating. Study participants who dined outside university cafeteria were more likely to cheat than those who dined in the university cafeteria. The odds of cheating among participants who engaged in substance abuse were significantly much higher than those who did not.

Other predictors significantly associated with cheating included absenteeism from lecture class, amount of pocket money/income and going to private high school: attendance at private high school was associated with significantly more cheating than attendance at a governmental high school. This might be due to the fact some private high schools in Ethopia are completely business oriented and do not let their students worry about honor codes. However, contrary to our findings, some studies found no correlation between type of high school attended and current cheating behavior [14]. Our study also found that the odds of cheating among students who didn’t attend lecture class were significantly higher than those students who never missed lecture classes. Also, those students who used one or more substances were more likely to cheat compared to those students who didn’t.

Our study didn’t show a significant association between cheating and gender, background, parental education level, interest on field of study and academic achievements. Consistent with our finding, few studies documented no association between cheating and age, gender, background [14]. Contrary to our finding, several studies document an association between cheating and gender, grade point averages, background [10,13,14,17].

The major limitation of the study is that a descriptive cross-sectional study with quantitative components can describe but not explain behavior. In addition, this type of study cannot establish causality between cheating and potential predictors. All data on cheating behaviours were self-reported and subjected to recall bias. Moreover, under-reporting of cheating behaviour is possible. Due to honor codes, wanting to feel socially desirable, and not wanting to report cheating in friends, some participants might not have answered the study questions accurately. So, we expect the prevalence of cheating to be even higher than the reported. Despite the limitations of the survey, the finding shows that too many participants do cheat and thus indicate a need to develop academic culture that promotes integrity and deals openly with the problem of cheating. The high response rate (89%) further strengthens the conclusiveness of this result. Furthermore, the findings incite various issues that could generate interest in investigating in-depth the reasons for cheating.

## Conclusions

This study revealed that one in five students of Hawassa University College of medicine and health science cheats even though the majority regarded it as an unacceptable behavior. It also found that current cheating in the university is very strongly associated with cheating in high school, cheating on the entrance examination, field of study, year of study and passive cheating. Cheating was also found to be strongly associated with dining outside university cafeteria and substance abuse. Cheating was significantly associated with absenteeism from lecture class, attendance at private school and amount of pocket money. On the other hand gender, age, background, parental education level, liking field of study, were not significantly associated with cheating at the university. Cheating at a university could well be a predictor of workplace dishonesty and workplace dishonesty among medical and health professionals has dire consequences for human life. At a wider level, it negatively affects social values and weakens the economy because it produces health care professionals with questionable credentials.

### Recommendations

Our findings should be help to establish interventions that discourage cheating and positive attitudes toward cheating at the Hawassa University College of Medicine and Health Science as well as other higher institutions. Institutions that train health care professional should develop culture that promote integrity and openly address the problem of cheating. Recommendations provided by other studies on cheating include; screening medical students for admission based on ethical maturity and not just on high grades [8] and the teaching of medical ethics in small discussion groups that focus on daily ethical dilemmas faced by students [27]. Our findings have also important policy implications: enforcing the academic integrity of an institution of higher education should in turn force high schools to examine their policies on cheating. A stricter policy against cheating in high school would then reduce cheating at the university. Finally, increasing the level of supervision during entrance examination, mandating attendance at lectures and discouraging substance use among students are also likely to reduce cheating.

## Competing interests

The authors declare that they don’t have any competing interests.

## Authors’ contributions

A.A.D conceived the original idea, drafted the proposal, involved in all implementation stages of the project, and write up; A.B reviewed the proposal and involved in all implementation stages of the project and write up. Both authors read and approved final version of the manuscript.

## Pre-publication history

The pre-publication history for this paper can be accessed here:

http://www.biomedcentral.com/1472-6920/14/89/prepub
